# The Opioid-Sparing Effect of Perioperative Dexmedetomidine Combined with Oxycodone Infusion during Open Hepatectomy: A Randomized Controlled Trial

**DOI:** 10.3389/fphar.2017.00940

**Published:** 2018-01-04

**Authors:** Benhou Zhang, Guifang Wang, Xiaopeng Liu, Tian-Long Wang, Ping Chi

**Affiliations:** ^1^Department of Anesthesiology, Xuan Wu Hospital, Capital Medical University, Beijing, China; ^2^Department of Anesthesiology, Beijing You An Hospital, Capital Medical University, Beijing, China; ^3^Department of Medical Insurance, Beijing You An Hospital, Capital Medical University, Beijing, China

**Keywords:** dexmedetomidine, oxycodone, hepatectomy, patient-controlled analgesia, nausea and vomiting

## Abstract

**Background:** A large right subcostal incision performed by open hepatectomy is associated with significant post-operative pain and distress. However, post-operative analgesia solutions still need to be devised. We investigated the effects of intra- and post-operative infusion of dexmedetomidine (Dex) combined with oxycodone during open hepatectomy.

**Methods:** In this prospective, randomized and double-blind investigation, 52 patients undergoing selective open hepatectomy were divided into Dex group (DEX infusion at an initial loading dose of 0.5 μg⋅kg^-1^ over 10 min before intubation then adjusted to a maintenance dose of 0.3 μg⋅kg^-1^⋅h^-1^ until incision suturing) or control (Con) group (0.9% sodium chloride was administered). Patient-controlled analgesia was administered for 48 h after surgery (Dex group: 60 mg oxycodone and 360 μg DEX diluted to 120 ml and administered at a bolus dose of 2 ml, with 5 min lockout interval and a 1 h limit of 20 ml. Con group: 60 mg oxycodone alone with the same regimen). The primary outcome was post-operative oxycodone consumption. The secondary outcomes included requirement of narcotic and vasoactive drugs, hemodynamics, incidence of adverse effects, satisfaction, first exhaust time, pain intensity, and the Ramsay Sedation Scale.

**Results:** Post-operative oxycodone consumption was significantly reduced in Dex group from 4 to 48 h after surgery (*P* < 0.05). Heart rate in Dex group was statistically decreased from T1 (just before intubation) to T6 (20 min after arriving at the post-anesthesia care unit), while mean arterial pressure was significantly decreased from T1 to T3 (during surgical incision; *P* < 0.05). The consumption of propofol and remifentanil were significantly decreased in Dex group (*P* < 0.05). The VAS scores at rest at 1, 4, and 8 h and with cough at 24, and 48 h after surgery were lower, the first exhaust time were shorter, satisfaction with pain control was statistically higher and the incidence of nausea and vomiting was less in Dex group than in Con group (all *P* < 0.05).

**Conclusion:** The combination of DEX and oxycodone could reduce oxycodone consumption and the incidence of nausea and vomiting, enhance the analgesic effect, improves patient satisfaction and shorten the first exhaust time.

## Introduction

Hepatectomy is the optimal treatment for patients with benign liver tumors, primary or metastatic liver malignancies, and some biliary diseases (Beard et al., 2000; Ramos et al., 2003; Poon et al., 2004). Over the past few decades, the prevalence of liver resection surgery improvements has significant increased due to the better hemostatic control, improvement in surgical technique, and wider indications for liver resection. Because of the location of the liver and the tendency of bleeding during resection, operation is typically performed through a big right subcostal incision. However, severe post-operative pain and distress which may result in delayed mobilization and physiotherapy, respiratory complications, and prolonged hospital stay after surgery with the large subcostal incision (Morrison et al., 2003).

In recent decades, continuous or bolus infusions of opioids have been widely used in patient-controlled analgesia after liver resection. However, an increase in analgesic-related side effects, such as sedation, respiratory depression, pruritus, hallucinations and post-operative nausea and vomiting (PONV) has also been reported (Aydogan et al., 2015; Khan et al., 2015; Allen et al., 2017). It is a challenge for anesthesiologists to perform a technique that could control sedation, analgesia, muscle relaxation, hemodynamic stability during the liver resection, and opioid-sparing analgesia after the surgery.

Therefore, one of the most beneficial interventions need to investigate is the reduction of pain intensity and opioid consumption by an adjunct drug combined with an opioid. Some adjunct drugs have the protective and opioid-sparing effects, such as ketamine, ketorolac, *N*-methyl-D-aspartate antagonists, and α_2_ adrenergic receptor agonists (Lin et al., 2009; White et al., 2012; Su et al., 2016). Dexmedetomidine (DEX), a highly selective α_2_ adrenergic receptor agonist, was used and studied as an important analgesic adjunct drug during the perioperative time of surgery. DEX has a more favorable pharmacokinetic profile than clonidine: α_2_:α_1_ specificity ratio, 1600:1 vs. 200:1, respectively; plasma half-life T½, 2–2.5 h vs. 9–12 h (Gil et al., 2009). it also showed many clinical benefits, such as an analgesic sparing and a sympathetic effect, sedation, analgesia, but without significant respiratory depression (Goodwin et al., 2013). DEX can be used safely for intra- or post-operative (Wang et al., 2014; Song et al., 2016), but the opioid-sparing effects of DEX infusion during an intra- and post-operative time in hepatectomy have not been studied.

Many studies have reported the opioid-sparing analgesic effects of DEX, either combination with opioids for a continuous infusion intraoperatively or post-operative analgesia (Su et al., 2016). However, no studies have concentrated on the analgesic effects of DEX and oxycodone during open abdominal surgery. This is the first study to evaluate its opioid-sparing analgesic following open liver resection surgery. DEX exerts its effects through α_2_-adrenoceptors (α_2_ R) and has a powerful affinity for this receptor (almost eight times greater than clonidine). Studies suggest that DEX activates α_2_R in the locus coeruleus of the brain and spinal cord and then cause the activation of potassium channel, facilitating Kþ efflux, and inhibition of voltage-gated Ca2þ channels, which is the main mechanism by which it exerts its anesthetic effects (Owesson et al., 2003).

The aim of this prospective study was to study the opioid-sparing analgesic effect of an infusion of DEX and adverse events for the first 48 h after open liver resection.

## Materials and Methods

### Patients

A prospective, randomized, and double-blinded investigation was performed on patients underwent elective open liver resection in Beijing You An Hospital, Capital Medical University. The study protocol was approved by the Research Ethics Committee of Beijing You An Hospital, Capital Medical University in Beijing, China [Ref: (2016)10]. The trial was registered at chictr.org at the beginning of the study (Ref: ChiCTR-IPR-17011001, March 2017), and written informed consent was obtained from all patients. Patients who undergoing elective open liver resection for malignant and benign liver lesions under general anesthesia from March 2017 to August 2017 were enrolled in this study if they met the following inclusion criteria: age between 18 and 70 years, American Society of Anesthesiologists (ASA) grade I–II. Exclusion criteria included laparoscopic liver resection, emergency operation, blood loss greater than 1000 mL, ASA physical status 3 or greater, Child-Pugh class C, allergy to any drug/agent used in the study, pre-existing chronic pain, opioid dependence, a left ventricular ejection fraction of <35%, greater than first-degree atrioventricular block, chronic renal dysfunction requiring replacement therapy, altered mental status, inability to provide adequate informed consent, or patient refusal, use of psychiatric medications, pregnant or lactating.

Before the operation, patients were randomized into a control (Con) group or a Dex group (30 per group). The randomization sequence without stratification was generated by a computer, and sealed with consecutively numbered envelopes. Treatment allocation was revealed by opening the envelope by nurses on the morning of surgery, the nurses were responsible for preparing DEX or saline placebo with the same 50 ml syringe and the post-operative patient controlled intravenous anesthetic drugs. And then the anesthesiologist administered the drug in a blind mode. Patients and investigators were all blinded to group allocation until the final statistical analysis was completed.

### Anesthesia

Oxygen saturation, electrocardiography, end-tidal CO_2_, arterial pressure and temperature were continuously monitored with an automated system (Philips IntelliVue MX800) when patients arrived at the operating room, then a peripheral intravenous catheters (G14) were placed before induction. A double lumen catheter (Foshan Special Medical Co., Ltd., China) was inserted in the right internal jugular vein for central venous pressure (CVP) measurement and a radial artery cannula (Becton Dickinson Infusion Therapy Systems, United States) was inserted for arterial pressure measurement and arterial blood gas test. Surface electrodes for the bispectral index (BIS; Aspect Medical System, Norwood, MA, United States) were placed to the patient’s forehead. The urine output was monitored through a 14 Fr catheter after the induction of anesthesia. A forced-air warming device (Patient Warming System, Warm Touch, United States) was used in both groups to maintain normothermia.

Patients in Dex group received intravenous DEX (0.5 μg⋅kg^-1^; Precedex; Aibeinin^®^, Inc., Henrui Pharmaceutical, China) over 10 min before endotracheal intubation, then adjusted to 0.3 μg⋅kg^-1^⋅h^-1^ from the 50 ml syringe until incision suturing. While in the Con group, an initial loading dose of 0.9% sodium chloride was administered as placebo followed by a maintenance dose from the same syringe at a relevant rate to the Dex group. All patients were induced with the same agents: Oxycodone (0.2 mg⋅kg^-1^; Hamol LIMITED, United Kingdom), propofol (2 mg⋅kg^-1^), and cisatracurium (0.2 mg⋅kg^-1^). After tracheal intubation, anesthesia was maintained with an infusion of propofol 4–8 mg⋅kg^-1^⋅h^-1^, a remifentanil (0.05–0.4 μg⋅kg^-1^⋅min^-1^) and cisatracurium (1–2 μg⋅kg^-1^⋅min^-1^) infusion according to clinical needs. Mean arterial pressure (MAP) was kept at a target of 65 mm Hg. Ringer’s lactate solution and gelofusine were used during the surgery. Positive pressure ventilation were maintained with endotracheal intubation to achieve an end-tidal pressure of CO_2_ of 30–40 mmHg. The rate of remifentanil and propofol were adjusted according to the hemodynamic stability, and depth of anesthesia was evaluated using the bispectral index at a target around 50 during surgery.

The baseline heart rate (HR) and MAP were defined as the mean of the two lowest measurements recorded before the induction of anesthesia. During the operation, tachycardia and bradycardia were defined as *a* > 30% increase from baseline or HR < 45 beats per minute (bpm) and were treated using esmolol (10–20 mg) or atropine (0.2–0.5 mg), respectively. Hypertension was defined as *a* > 30% increase from baseline and was treated by increasing the remifentanil; and if hypertension persistent after remifentanil treatment, urapidil (10–15 mg) was administered. Hypotension was defined as *a* > 30% decrease from baseline and was treated by decreasing the remifentanil, and if persistent, by incensement the rate of fluid, administration of phenylephrine (20–50 μg) or ephedrine (6–12 mg) according to the clinical need. DEX and cisatracurium were discontinued approximately 30 min before completion of the surgery, while all patients received tropisetron (5 mg) and oxycodone (0.1 mg⋅kg^-1^). Neostigmine and atropine were given to reverse residual neuromuscular block at the end of surgery. All patients were transferred to the post-anesthesia care unit (PACU) for further observation after extubation in the operating room.

### Post-operative Analgesia Management

One day before surgery, the patients were instructed to use the PCA pump (Apon, China) and a 10 cm Visual Analog Scale (VAS) score (0–3 mild pain; 4–6 moderate pain; 7–10 severe pain). PCA protocol in Con group (without a background infusion) consisted of 60 mg oxycodone diluted to 120 ml and administered at a bolus dose of 2 ml, with 5 min lockout interval and a 1 h limit of 20 mL, while in Dex group was 60 mg oxycodone and 360 μg Dex diluted to 120 ml with the same regimen as those in Con group after arriving at the PACU.

Patients were encouraged to press the pump button when they experienced pain with cough at a severity of VAS score > 4. If the pain control was not satisfied, then rescue analgesia (40 mg of parecoxib or 100 mg of tramadol by intravenous injection) was used. The PCIA pump will be stopped on the second post-operative day, and PONV will be treated with 5 mg of tropisetron according to the patient’s request.

### Data Collection

Age and body mass index (BMI; calculated as weight (kg)/[height (m)]^2^), ASA, Child–Pugh scores and operative time were collected from the patient charts. Child–Pugh scores employs five clinical measures of liver disease. Each measure is scored 1–3, with 3 indicating most severe derangement. Chronic liver disease is classified into Child–Pugh class A to C, employing the added score from above. Class A, 5–6; Class B, 7–9; Class C, 10–15 (Cholongitas et al., 2005). Duration of surgery was measured from the surgical incision to the closure of the skin. HR and MAP were got from the Philips IntelliVue monitor at the following points: arrival at the operating room (T0), just before intubation (T1), after intubation (T2), during surgical incision (T3), on extubation (T4), on arrival at the PACU (T5), and 20 min after arriving at the PACU (T6). The requirement of narcotic and vasoactive drugs, estimated blood loss, fluid requirements, and complications were recorded. The PACU stay time was also recorded based on the Aldretes criteria (Aldrete, 1995).

The cumulative amount of self-administered oxycodone was recorded until 48 h after the surgery. Pain at rest and with cough was assessed with the VAS, where 0 denotes no pain and 10 denotes the worst pain experienced. The Ramsay Sedation Scale was also used to assess the sedation state, where 1 = anxious, agitated, or restless; 2 = cooperative, oriented, and tranquil; 3 = responds to command; 4 = asleep but with brisk response to a light glabellar tap or loud auditory stimulus; 5 = asleep, sluggish response to a light glabellar tap or loud auditory stimulus; and 6 = asleep, no response to stimuli. PONV scores from 1 to 4 (1 = without nausea and vomiting, 2 = nausea without vomiting, 3 = less than two times vomiting, 4 = severe vomiting more than two times) (Apfel et al., 1999). These were evaluated at 1, 4, 8, 12, 24, 48 h after surgery. Patients were asked to rank their satisfaction with pain control according to the following scale: 1 = not satisfied, 2 = moderately satisfied, 3 = satisfied, 4 = very satisfied at 48 h after the PCA pump was removed (Harms et al., 2007). The number of rescue analgesia and adverse effects (such as hypertension, hypotension, bradycardia, tachycardia, respiratory depression, liver failure, and death within 48 h after surgery) was also recorded.

### Statistical Analysis

Data are expressed as the mean ± SD, percentage (%), and numbers (n). The distribution of variables was assessed by the Kolmogorov–Smirnov. Homogeneity of variance was determined using Levene’s tests. Inter-group comparisons were performed using repeated-measures analysis of variance. The non-parametric Mann–Whitney–Wilcoxon Test was used for variables that were not normally distributed. Categorical data were analyzed using Fisher’s exact tests or chi-squared tests. Probability (*P*) values of <0.05 were considered statistically significant. Statistical analysis was performed using SPSS for Windows Version 21.0 (SPSS Inc., Chicago, IL, United States).

## Results

### Baseline Characteristics

The CONSORT demographics are described in a patient enrollment flow diagram. Sixty patients who underwent liver surgery were screened from March 2017 to August 2017. Eight patients were excluded because of not meeting the inclusion criteria: five patients refused to participate, the blood loss of three patients was more than 1000 ml. Finally, 52 patients were finally enrolled in the primary analysis and randomly divided into two groups: 26 patients in Dex group and 26 patients in Con group. No patients failed to follow-up.

Baseline characteristics of the 52 patients were comparable between the groups. As shown in **Table 1**, there were no significant differences in age, weight, height, BMI, sex, ASA grade, Child–Pugh grade, tumor type and history of comorbidity (all *P* > 0.05; **Table 1**).

**Table 1:** Perioperative characteristics and data of patient in the two groups.

	Dex (*n* = 26)	Con (*n* = 26)	*P*-values
**Preoperative**			
Age (year)	41.12 ± 8.468	43.12 ± 8.842	0.409
Weight (kg)	62.36 ± 7.57	61.79 ± 7.17	0.781
Height (cm)	169.85 ± 7.15	168.38 ± 6.99	0.46
BMI (kg⋅m^-2^)	21.62 ± 2.19	21.77 ± 1.93	0.789
Sex and males	17 (65%)	15 (58%)	0.569
ASA I/II	21/5	20/6	0.734
Child-Pugh score A/B^#^	23/3	24/2	0.884
Malignant/benign disease	23/3	24/2	0.884
**Preoperative comorbidity, *n* (%)**			
Hypertension	4 (15%)	5 (19)	0.773
Diabetes mellitus	2 (8%)	3 (12%)	
Arrhythmia	2 (8%)	3 (12%)	
**Intraoperative**			
Operating time (min)	170.81 ± 13.30	175.65 ± 18.75	0.288
Duration of anesthesia (min)	216.19 ± 12.00	221.15 ± 20.02	0.284
Bispectral index	44.92 ± 3.89	44.38 ± 3.68	0.610
Estimate blood loss (ml)	446.15 ± 147.60	526.23 ± 187.35	0.093
Fluids (ml)	2706.96 ± 163.98	2741.31 ± 159.88	0.448
Urine output (ml)	586.46 ± 147.46	516.38 ± 108.06	0.056
Propofol dosage (mg⋅kg^-1^⋅h^-1^)	4.12 ± 0.23	5.47 ± 0.35^∗^	0.000
Remifentanil dosage (μg⋅kg^-1^⋅min^-1^)	0.26 ± 0.02	0.35 ± 0.03^∗^	0.000
Cisatracurium dosage (μg⋅kg^-1^⋅min^-1^)	1.54 ± 0.09	1.55 ± 0.09	0.444
**Post-operative**			
PACU time (min)	48.73 ± 11.77	45.10 ± 11.54	0.232
Rescue analgesic requirement	3 (11.54%)	9 (34.62%)^∗^	0.048
First exhaust time (h)	54.04 ± 6.88	61.38 ± 11.42^∗^	0.007
**Post-operative adverse events**			
Hypertension	6 (23.08%)	7 (26.92%)	0.749
Hypotension	2 (7.69%)	1 (3.85%)	0.552
Tachycardia	3 (11.54%)	5 (19.23%)	0.442
Bradycardia	3 (11.54%)	2 (7.69%)	0.638

*Continuous data are reported as mean ± SD, and categorical data are expressed as counts and percentages. ^#^Child-Pugh score employs five clinical measures of liver disease. Each measure is scored 1–3, with 3 indicating most severe derangement. Chronic liver disease is classified into Child–Pugh class A to C, employing the added score from above. Class A, 5–6 points; Class B, 7–9 points; Class C, 10–15 points. BMI, body mass index; ASA, American Society of Anesthesiology; PACU, post-anesthesia care unit. ^∗^*P* < 0.05 vs. Con group.*

### Intraoperative Variables

**FIGURE 1:**
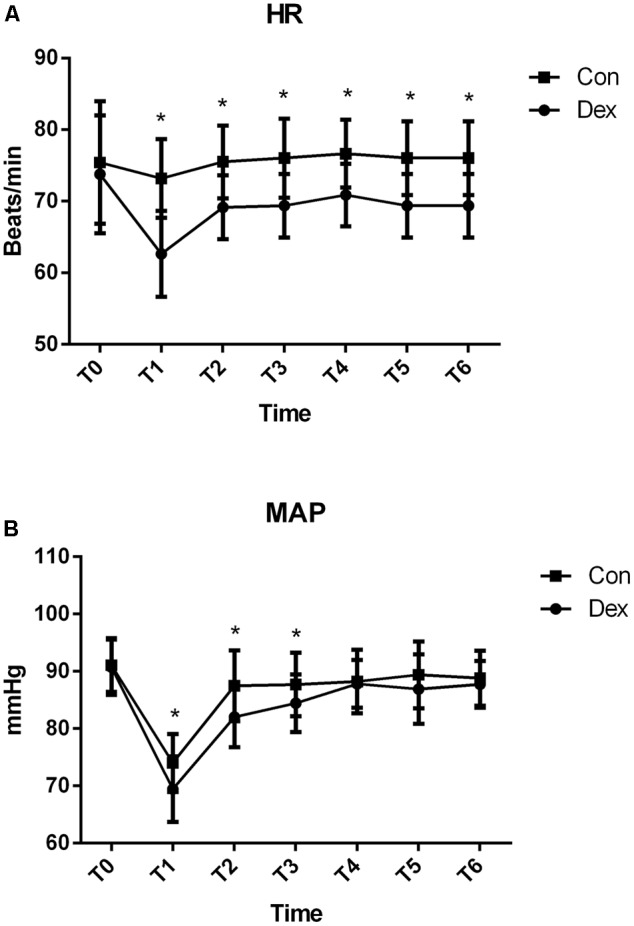
Hemodynamics monitoring between the two groups. **(A)** Comparison of heart rates (HR; beats/min) and **(B)** Comparison of mean arterial pressure (MAP; mmHg) in the two groups at different time points. T0, arrival at the operating room; T1, just before intubation; T2, after intubation; T3, during surgical incision; T4, on extubation; T5, on arrival at the PACU; T6, 20 min after arriving at the PACU. Baseline HR and MAP were similar between the two groups (*P* > 0.05). Compared with Con group, HR in Dex group was statistically decreased from T1 to T6, while MAP was significantly decreased from T1 to T3 (*P* < 0.05). ^∗^*P* < 0.05.

**FIGURE 2:**
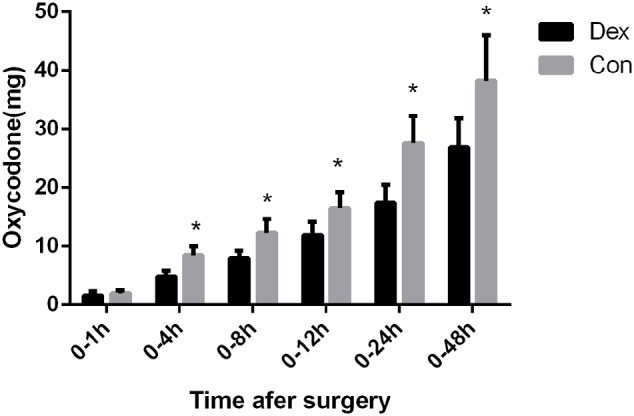
Post-operative consumption of PCA oxycodone in the two groups. The total dosage of oxycodone was significantly lower in Dex group than in Con group at 4, 8, 12, 24, and 48 h after surgery (*P* < 0.05), ^∗^*P* < 0.05.

AS displayed in **Figure 1**, baseline HR and MAP were similar between the two groups (*P* > 0.05; **Figure 1**). Compared with Con group, HR in Dex group was statistically decreased from T1 to T6, while MAP was significantly decreased from T1 to T3 (*P* < 0.05; **Figure 2**), but MAP was similar in two groups from T4 to T6 (*P* > 0.05; **Figure 2**).

As shown in **Table 1**, there were no statistically differences in operating time, duration of anesthesia, bispectral index, estimate blood loss, fluids infusion, urine output, and consumption of cisatracurium (*P* > 0.05; **Table 1**). The consumption of propofol and remifentanil were significantly decreased in Dex group Compared with Con group (*P* = 0.000; **Table 1**).

The patients required for intraoperative vasoactive drugs such as esmolol (3 vs. 9, *P* = 0.048), and urapidil (4 vs. 11, *P* = 0.032) were less in Dex group than Con group. While the usage of atropine, ephedrine, and phenylephrine were similar between the two groups (**Table 2**).

**Table 2:** The usage of vasoactive drugs during surgery.

	Dex (*n* = 26)	Con (*n* = 26)	*P*-values
Atropine	2 (7.69%)	1 (3.85%)	0.552
Ephedrine	4 (15.38%)	3 (11.54%)	0.685
Phenylephrine	1 (3.85%)	2 (7.69%)	0.552
Esmolol	3 (11.54%)	9 (34.62%)^∗^	0.048
Urapidil	4 (15.38%)	11 (42.31%)^∗^	0.032

*Continuous data are reported as mean ± SD, and categorical data are expressed as counts and percentages. ^∗^*P* < 0.05 vs. Con group.*

### Post-operative Variables

**FIGURE 3:**
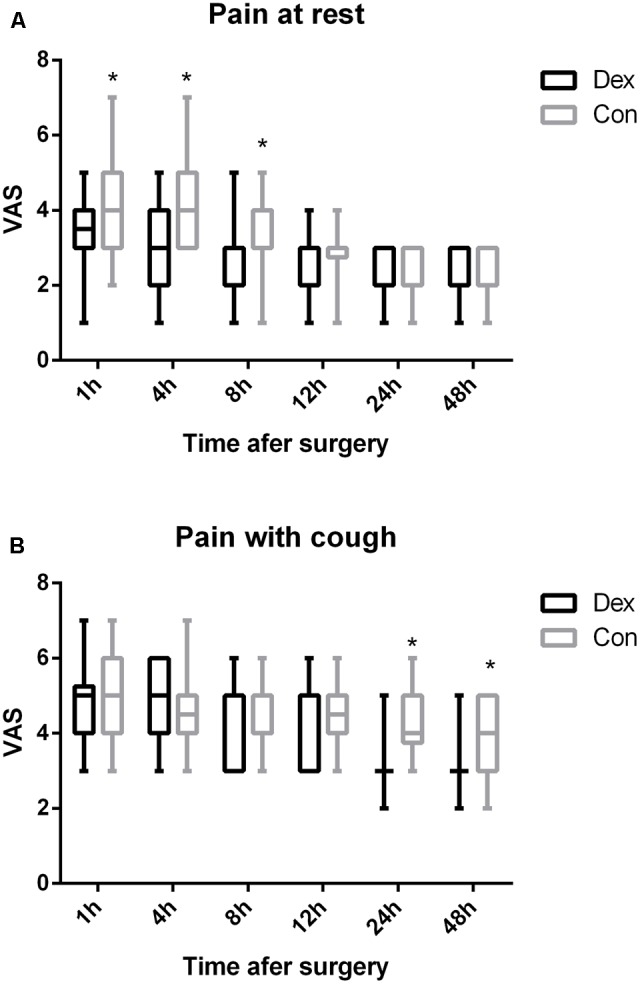
**(A,B)** Post-operative pain at rest or with cough were assessed with a visual analog scale (VAS) out of 10. 0, no pain; 10, the worst pain. The VAS scores at rest at 1, 4, and 8 h post-operative and with cough at 24, and 48 h after surgery were statistically lower in Dex group compared with Con group (*P* < 0.05). ^∗^*P* < 0.05.

**FIGURE 4:**
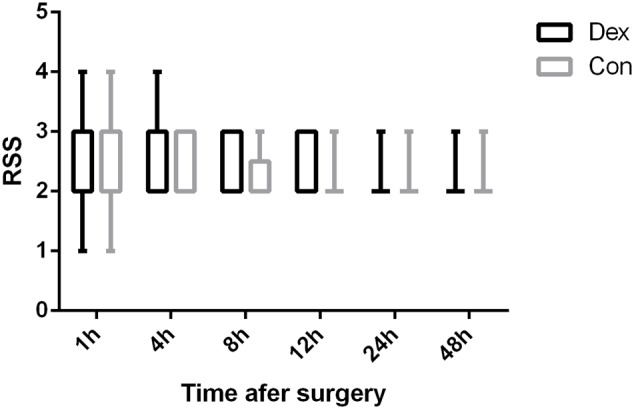
Comparison of patients’ sedation (Ramsay sedation scale [RSS]). There were no significant differences in RSS within the first 48 h after surgery between the two groups (*P* > 0.05). 1, anxious, agitated, or restless; 2, cooperative, oriented, and tranquil; 3, responds to command; 4, asleep but with brisk response to a light glabellar tap or loud auditory stimulus; 5, asleep, sluggish response to a light glabellar tap or loud auditory stimulus; and 6, asleep, no response to stimuli.

As displayed in **Figure 2**, the total dosage of oxycodone was significantly lower in Dex group than in Con group at 4, 8, 12, 24, and 48 h after surgery (*P* < 0.05, **Figure 2**). The VAS scores at rest at 1, 4, and 8 h post-operative and with cough at 24, and 48 h after surgery were statistically lower in Dex group compared with Con group (*P* < 0.05, **Figure 3**), while the VAS scores at other time were similar between the two groups. There was no significant difference in RSS within the first 48 h after surgery between the two groups (*P* > 0.05, **Figure 4**).

**FIGURE 5:**
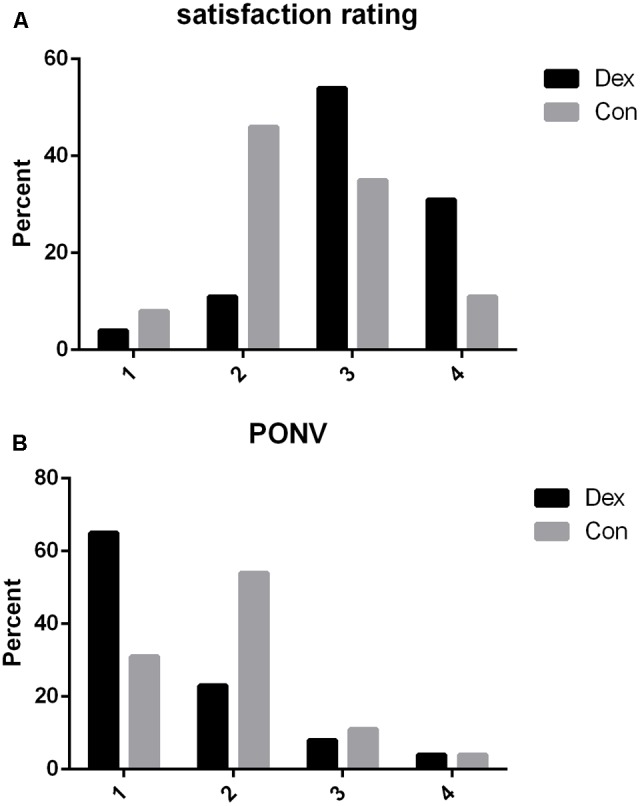
**(A)** Post-operative satisfaction rating to pain control within 48 h after surgery. 1, not satisfied; 2, moderately satisfied; 3, satisfied; 4, very satisfied. Satisfaction with pain control was statistically higher in Dex group than in Con group (*P* = 0.033). **(B)** Post-operative nausea and vomiting rating within 48 h after surgery in two groups. 1, without nausea and vomiting; 2, nausea without vomiting; 3, less than two times vomiting; 4, severe vomiting more than two times. The incidence of PONV was less in Dex group than in Con group (*P* = 0.031).

More patients required rescue analgesia in Con group compared with Dex group within 48 h after surgery (3 VS. 9, *P* = 0.048, **Table 1**). The first exhaust time (54.04 ± 6.88 VS. 61.38 ± 11.42 min, *P* = 0.007) were earlier in Dex group than in Con group after surgery (**Table 1**). Satisfaction with pain control was statistically higher in Dex group than in Con group (*P* < 0.05, **Figure 5A**), while the incidence of PONV was less in Dex group than in Con group (*P* < 0.05, **Figure 5B**).

As shown in **Table 1**, PACU time and the incidence of other main adverse events such as hypertension, hypotension, tachycardia, and bradycardia are similar in two groups (*P >* 0.05, **Table 1**). No respiratory depress, liver failure, and no patients died within 48 h after surgery (**Table 1**).

## Discussion

In this prospective, randomized, and double-blinded trial, we found that DEX plus oxycodone as PCA in patients who underwent open liver resection could reduce both the total consumption of oxycodone from 4 to 48 h and the pain intensity after surgery. The HR and MAP, the requirement of propofol, remifentanil, esmolol, and urapidil during surgery were significantly decreased in Dex group. The incidence of PONV and requirement of post-operative rescue analgesia was decreased, the first exhaust time was shortened, while the satisfaction to pain control was improved in Dex group than in Con group.

Epidural analgesia has been considered superior to intravenous analgesia for post-operative pain relief in patients recovering from major upper abdominal operations (Werawatganon and Charuluxanun, 2005). However, the use of patient epidural analgesia after hepatectomy is still a subject to debate. Because epidural analgesia can lead to serious complications, such as epidural hematoma, it may be contraindicated when post-operative coagulopathy is expected (Matot et al., 2002; Cook et al., 2009). Any increase in epidural bleeding risk is an important consideration in liver resection, where the incidence of post-operative coagulopathy may be as high as 47% (Shontz et al., 2009) with the degree of coagulopathy correlating with the extent of liver resection (Oguro et al., 1993). In addition, epidural methods take time to induce anesthesia and may not function adequately in up to 30% of patients (McLeod et al., 2001). Compared to epidural analgesia, patient satisfaction with i.v. PCA is higher (Werawatganon and Charuluxanun, 2005). These were reasons we chose patient controlled intravenous analgesia (PCIA) for patients underwent hepatectomy in this study.

In the past, morphine is the opioid that most commonly used for post-operative pain, because it is economical and easy to manage, but there is a trend toward the use of oxycodone. Oxycodone is one of the most widely used opioids for pain management (Kokki et al., 2012), it is a semi-synthetic opioid that may be an agonist of the central and peripheral kappa-as well as mu-opioid receptors (Lalovic et al., 2006; Nielsen et al., 2007), is superior in the treatment of visceral pain than other opioids (Staahl et al., 2007). Previous studies have shown good efficacy of oxycodone in managing post-operative pain (Blumenthal et al., 2007; Koch et al., 2008). The analgesic efficacy of oxycodone is 1.6 times higher than that of morphine. Now Oxycodone is replacing morphine as a first-choice opioid in several countries. However, some side effects such as nausea, vomiting, and respiratory depression have also been reported (Koch et al., 2008). A multimodal analgesia that could enhance analgesia and reduce the requirement for opioids would be productive (Su et al., 2016).

Dexmedetomidine has the anxiolytic and moderate analgesic effects, it is thought through the binding of central and peripheral α2 receptors. Compared with other sedatives (Yoshida et al., 2009), DEX causes light sedation and is less likely to cause respiratory depression (Venn et al., 1999). DEX can be effective in clinical analgesic treatment and reduce the consumption of opioids (Lin et al., 2009; Ren et al., 2015; Su et al., 2016). Bhana et al. (2000) have suggested that the onset time for DEX is approximately 15 min, with a distribution half-life of approximately 6 min and an elimination half-life of approximately 2 h. The recommended loading dose of DEX is 0.5 to 1 μg⋅kg^-1^. Su et al. (2016) found that DEX infusion at 0.5 μg⋅kg^-1^ for 10 min, then adjusted to 0.3 μg⋅kg^-1^⋅h^-1^ until incision suturing and 0.02 μg⋅kg^-1^⋅h^-1^ in combination with opioids for intravenous PCA led to a reduction in both the pain score and opioid consumption. In this study, we chose 0.5 μg⋅kg^-1^ loading dose then adjusted to 0.3 μg⋅kg^-1^⋅h^-1^ continuous infusion until incision suturing and combination with 360 μg DEX in PCA pump. We found that oxycodone consumption decreased (from 4 to 48 h after surgery), the VAS was lower (1, 4, and 8 h at rest and at 24, and 48 h with cough after surgery), and the rate of analgesic satisfaction was higher in Dex group compared with those who received only oxycodone, while the RSS during the first 48 h after surgery was similar in the two groups. Those indicate that DEX is an excellent choice as an adjuvant drug for PCA in patients who undergo hepatectomy.

Most common side effects related with DEX are hypotension and bradycardia (Bekker et al., 2013; Guen et al., 2014). In our study, the incidence of hypotension and bradycardia intra- or post-operative was not significantly different between the two groups. On the contrary, we found that more patients required esmolol, and urapidil in Con group. The result was consistent with the report of Su et al. (2016). This could be due to the antisympathetic action of DEX. Worrying about the decrease of liver function, we used a lower dose for continuous and bolus infusion than the recommendations of the DEX manufacturer and the previous study. Maybe this is a possible reason for this difference (Guen et al., 2014).

Post-operative nausea and vomiting are common adverse effects due to opioids after surgery, which are caused by opioids by stimulating the chemoreceptor trigger zone. PONV decrease patient overall satisfaction, and may also worsen the severity of incisional pain. Many patients have reported that post-operative vomiting is more unpleasant than post-operative pain (Macario et al., 1999). In our study, the incidence of PONV was lower in Dex group, partly because of the lower dose of oxycodone used for pain control in the PCA pump. Maybe that was one of the reasons why patient satisfaction score was higher in Dex group than in Con group, other reasons possibly contain lower VAS, and less PCA bolus.

In our study, a new finding was the helpful effect about DEX on bowel function recovery. The first exhaust time were shorter in the DEX group than in Con group. Opioids suppress the intestinal transit and the bowel movement, and opioid receptor antagonist was demonstrated to improve gastrointestinal recovery (Berger et al., 2015). One explanation may be that the opioid-sparing analgesic effects of DEX reduced the usage dose of oxycodone. This will be confirmed in our future study, because the bowel function recovery time is very important indicator and closely related to the length of hospital stay.

There were several limitations in present study. First, this study was performed at one center. A multi-center trial through Investigate more various populations from different center would address more clinical outcome parameters in the future. Second, in the current study, DEX was administered 0.5 μg⋅kg^-1^ loading dose before intubation and adjusted the rate to 0.3 μg⋅kg^-1^⋅h^-1^ during the surgery. However, we did not monitor the serum concentration of DEX at any time point. And we will investigate the effects of other doses in the future. Third, in the PCA pump, the total dosage of both oxycodone and DEX were fixed, and were not calculated with patients’ body weight. However, this is a random design, there were no differences in the body weight between two groups. Different dosages of DEX and oxycodone per weight will be further investigated. Lately, the size of the cut-off liver was not measured during the surgery. However, the duration of surgery and anesthesia was similar between the two groups.

## Conclusion

The combination of DEX and oxycodone for PCA after hepatectomy could reduce oxycodone consumption and the incidence of PONV, enhance the analgesic effect, improves patient satisfaction and shorten the first exhaust time during the open liver surgery. Different dosages of DEX-oxycodone and a multi-center trials are required to further investigate during the perioperative period of hepatectomy.

## Author Contributions

BZ and GW contributed to this work equally. BZ, T-LW, and PC conceived and designed the trial. GW analyzed the data. BZ and GW collected the clinical data. BZ wrote the first draft of the paper. BZ and XL performed clinical anesthesia for the patients. T-LW and PC contributed equally to this trial and should be considered as co-corresponding authors.

## Conflict of Interest Statement

The authors declare that the research was conducted in the absence of any commercial or financial relationships that could be construed as a potential conflict of interest.
